# Clinical reliability of zirconium abutment in implant restorations in the English and Korean literature

**DOI:** 10.1186/s40902-018-0162-4

**Published:** 2018-09-10

**Authors:** Su-Been Yu, Bong-Gyu Song, Kyeong-Jun Cheon, Ju-Won Kim, Young-Hee Kim, Byoung-Eun Yang

**Affiliations:** 1 Department of Oral and Maxillofacial Surgery, Hallym University Sacred Heart Hospital, Anyang, South Korea; 2 Department of Oral Implantology, Graduate School of Clinical Dentistry, Hallym University, Chuncheon, South Korea; 3 Institute of Clinical Dentistry, Hallym University, Chuncheon, South Korea; 4 Department of Dentistry, National Police Hospital, Seoul, South Korea; 5 Department of Industrial Security, Hansei University, Gunpo, South Korea; 6 Department of Dentistry, Hallym University Sacred Heart Hospital, 22 Gwanpyeong-ro 170 beon-gil, Dongan-gu, Anyang, Gyeonggi-do 431-796 South Korea

**Keywords:** Zirconium abutment, Dental implant

## Abstract

**Background:**

This study aimed to evaluate the mechanical, biological, and esthetic stability of a zirconium abutment according to evidence-based dentistry.

**Main text:**

An electronic search was performed. Domestic studies were found using the keywords “zirconia abutments” and “zirconium abutment” in KMbase, KoreaMed, and the National Assembly Library, and international studies were found using the same keywords in PubMed. All identified studies were divided by evidence level from the viewpoint of the research type utilizing the evidence-based review manual. A total of 102 domestic studies (with Korean language) were found, and 9 of these studies were selected. In these nine studies, 3 had evidence level 3 and 6 had evidence level 4. A total of 97 international studies (with English language) were found, and 19 were selected. Among these 19 studies, 5 had evidence level 2 and 7 had evidence level 3, whereas the remainder had evidence level 4. According to the studies, zirconium abutments are mechanically, biologically, and esthetically stable, but the evidence level of these studies is low, and the follow-up duration is no longer than 5 years.

**Conclusions:**

All examined studies verified the mechanical stability of zirconium abutments for a period no longer than 5 years. Therefore, a long-term clinical observation is needed. Zirconium abutments are thought to be biologically stable, but they are not superior to titanium abutments. As the esthetic stability of such abutments had a low evidence level in the studies that examined here, a much higher evidence level is needed.

## Introduction

As the interest in the esthetic aspect of implant prosthesis increases, the importance of the esthetic element has been emphasized. It has been reported that ready-made titanium abutments leave a gray shade if the soft tissue is thin or if the gingival sulcus is shallow, and a non-esthetic outcome may be caused by the exposure of titanium in the gingival margin when a gingival recession occurs [1]. Alumina ceramic abutments have been developed to overcome the non-esthetic problem, but failed due to fracture caused by the brittleness of the alumina ceramic during functioning was reported. Since then, zirconium with high biological stability, an esthetic feature, and high mechanical stability has been used as an abutment [1]. According to the 2011 MFDS (Ministry of Food and Drug Safety, Republic of Korea) report, esthetic prosthetic treatment using zirconium prosthesis and implant abutment is on the spotlight, and over 12% of the high annual average growth rate has been shown in South Korea.

Zirconium abutments are widely used in South Korea, but it is not easy to find evidence of their clinical stability for long-term use by the Koreans. Glauser et al. reported that there was no fracture in the zirconium abutments in their study for an average of 49.2 months in single-tooth restoration [2]. Zembic et al. reported the long-term stability of zirconium abutments, showing a 96.3% success rate [3]. Additionally, the studies conducted by Canullo et al., Sailer et al., Zembic et al., and Nothdurft et al. reported a 100% success rate in zirconium abutment clinical practices [4–7]. All these studies, however, were conducted in Caucasian countries. Michael et al. reported that the maximum bite force was 725 N, and the average masticatory force was 262 N in natural teeth [8]. Ferrario et al. reported that the maximum bite force was around 700 N in healthy adult males and females [9]. In Korean research, however, Cho reported that the maximum bite force was 923.8 N on average [10], and Yoon reported that the average bite force was 744.5 N [11]. Koreans who favor hard foods such as kimchi, gakdugi, squid, and ribs deliver a strong masticatory force to the molar area [12]. Kim et al. examined the subjective food intake ability of the maximum occlusal force among Korean adults [13]. Considering the difference in diet and bite force between the Koreans and Caucasians, the stability of zirconium needs to be evaluated in the Koreans and the evidence level must be established.

According to the Straumann report 2013, Korea is the country where dental implant treatment is most performed [14]. The Korean National Health Insurance Corporation announced that it would expand the coverage of dental implants in patients aged 70 and older beginning in 2015 and those aged 65 and older in 2016. Only patients older than 65 are eligible, but Korea is the second country to add implant treatment to the national health insurance after Sweden. However, the zirconium abutment was not included in the health insurance. We have reviewed the validity of zirconium abutment through Korean and English literature and wanted to provide a basis for its use. This study intended to help increase the treatment stability by offering evidence to the dentist’s choice through the stability assessment of zirconium abutments, with a focus on evidence-based dentistry. The goal of this study is to review the status of clinical studies on the stability of zirconium abutments in papers in Korea and abroad and to refer to them for application in clinical medicine.

## Methods

A key question was set. The significant element in determining the key question included the patient/problem (P), intervention (I), comparison (C), and outcome (O) (PICO). The combination of these four major elements is “PICO asking questions.” According to PICO asking questions, “Does the zirconium abutment (P and I) have mechanical, biological, and esthetic stability (O) compared to the titanium abutment (C)?” was set as a key question.

### Search strategy

Literature was searched, using the keywords “zirconia abutment,” or “zirconium abutment,” in KoreaMed, KMbase, and National Assembly Electronic Library, which were recommended as domestic literature search sites by 「Evidence Literature Utilization Guidelines」 published by the Health Insurance Review and Assessment Service of Republic of Korea (2013a). Literature was searched using keywords including “zirconia abutment” and “zirconium abutment” in PubMed to compare the evidence levels of the stability of zirconium abutments in the domestic and international literature. The searched literature was selected by determining the inclusion and exclusion criteria based on the concept of evidence-based dentistry. The domestic literature included studies with Korean subjects and literature written in Korean. The scope of this study was clinical literature. Studies with international subjects, animal experiments, laboratory studies, expert commentaries, and scholarly literature were excluded.

### Stability assessment criteria and classification of the evidence level

#### Stability assessment criteria

In this literature, mechanical stability means that there are no problems of zirconium abutment fracture, abutment screw fracture, etc. To evaluate the biological stability, the conditions of the tissues around the zirconium abutments including the height of the bones, the plaque accumulation level in the soft tissue, the bleeding on probing, and the depth of the periodontal pocket were compared with the conditions of the tissues around the titanium abutments or around the healthy natural teeth. The esthetic stability was evaluated to be stable when the color and shape of the soft tissue surrounding the zirconium abutments were similar to those of the natural teeth.

#### Classification of the study type according to the evidence level

In this study, the grade criteria for clinical research literature suggested by the Health Insurance Review and Assessment Service were used. After the confirmation of the study type and contents that were selected based on Table 1, the evidence level was classified. According to the grade criteria for clinical research literature, the evidence level of systematic literature review targeting randomized controlled trials (RCTs) is the highest, and the evidence level of cross-sectional study, case series, before/after study, case report, and non-analytic study is the lowest. As such, the lower the grade is, the higher the level of evidence.

**Table 1:** Study type according to evidence level

Grade	Study type
1 (high)	Systematic literature review targeting RCTs (systematic review with/without meta-analysis)
2	Randomized controlled trial, systematic literature review targeting category 3
3	Quasi-RCT, cohort study, case-control study, observational/analytic study
4 (low)	Cross-sectional study, case series, before/after study, case report, non-analytic study

Source: Health Insurance Review and Assessment Service. Republic of Korea, 2013a

## Main text

**Fig. 1:**
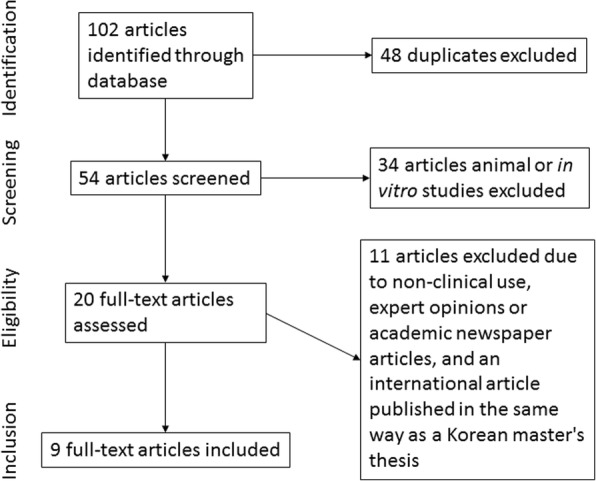
The flow chart of the search strategy of the Korean literature

As a result of the domestic literature search, a total of 102 kinds of literature were searched, including 28 kinds of literature at KoreaMed, 35 kinds of literature at KMbase, and 39 kinds of literature at the National Assembly Library. Of these 102 kinds of literature, 48 duplicate kinds of literature, 35 kinds of literature that fell under the exclusion criteria (including animal experiments and laboratory studies) based on a review of titles and abstracts, and three kinds of literature whose scope was not related to implant zirconium abutment were excluded. After a review of the full texts of the literature, six expert commentaries and one Master’s thesis that was published in an international journal with a different title were excluded. Finally, nine literature were selected (Fig. 1).

**Fig. 2:**
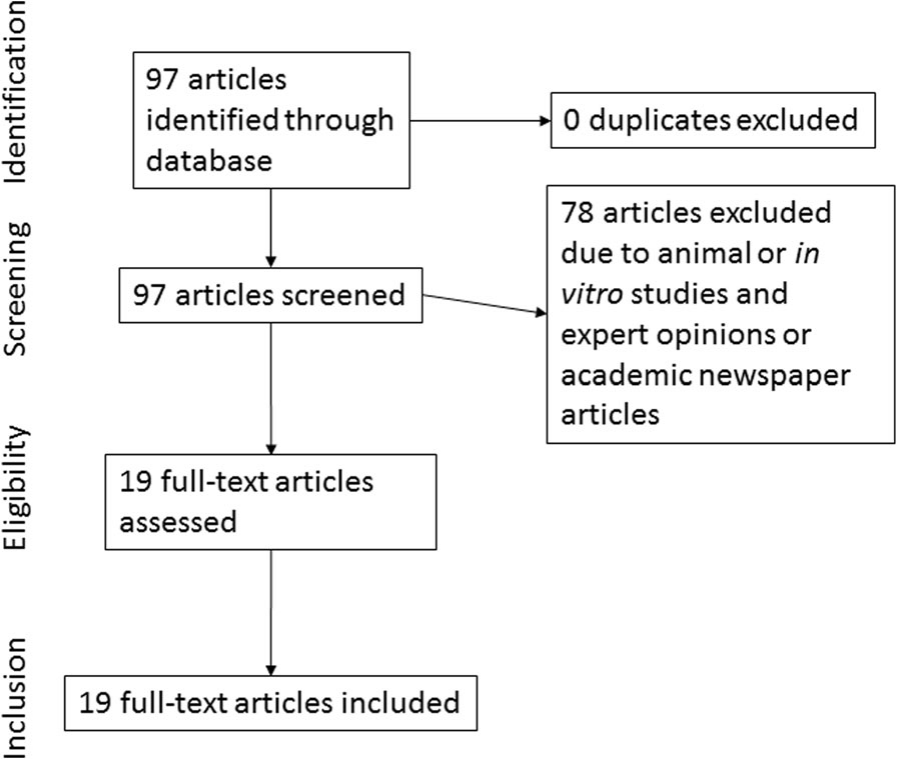
The flow chart of the search strategy of the international literature

International literature was searched at the Refomax Electronic Library of Hallym University Medical Center by linking with PubMed using keywords including “zirconium abutment” and “zirconia abutment.” The literature type was set to review, clinical trial, and case report, and a total of 92 kinds of literature were selected: 13 reviews, 37 clinical trials, and 42 case reports. Based on the titles and abstracts, animal experiments, laboratory studies, and studies not related to implant zirconium abutment were excluded, and only the literature whose full texts could be obtained from the electronic library of the Hallym University Medical Center were selected. Finally, five reviews, eight clinical trials, and six case reports were selected (Fig. 2).

After the confirmation of the study types of the domestic and international literature, the evidence levels were classified. The classification results are presented in Table 2. For the domestic literature, there was no systematic literature review targeting RCTs, RCTs, and systematic literature reviews, which fall under evidence levels 1 and 2, respectively. There were three cohort studies with evidence level 3, and 6 case reports with evidence level 4.

**Table 2:** Status of the evidence levels of domestic and international studies

Grade	Study type	Result (literature)	
		Domestic	International
1	Systematic literature review targeting RCTs (systematic review with/without meta-analysis)	0	0
2	RCT	0	0
	Systematic literature review targeting category 3	0	5
3	Quasi-RCT	0	0
	Cohort study	3	4
	Case-control study	0	3
	Observational, analytic study	0	0
4	Cross-sectional study	0	0
	Case series	0	1
	Case report	6	6
	Non-analytic study	0	0

In the international literature search process, there was a function for setting a literature type; thus, it was easy to classify the evidence level compared to the domestic literature search. About the evidence levels of international literature, there were five systematic literature reviews with evidence level 2, four cohort studies, and three case-control studies with evidence level 3, and 1 case series and 6 case reports with evidence level 4.

### Domestic literature

#### Evidence level 3

A summary of three domestic kinds of literature with evidence level 3 is presented in Table 3. In the studies conducted by Kim et al., regarding the stability of zirconium abutments, the survival rate was high regardless of the implant location and prosthesis type, and the number of prosthesis units and prosthesis types appeared to be significantly associated with the complication rate of zirconium abutments [15]. Also, the zirconium abutments were mechanically stable within the 5-year follow-up period. Bae et al. reported that the zirconium abutments in their study were biologically stable [16].

**Table 3:** Summary of domestic studies with evidence level 3

Author	Study type	Patient (person)/implant (unit)/restoration	Implant location	Prosthesis type	Zirconium abutment	F/U period	Outcome	Conclusion
Kim et al. [34]	Prospective cohort	213/611/328	Anterior/posterior	Single unit/multi-units with pontic	Alumina-toughened zirconium abutment (ZirAce)	3.6 years (1–12.8 years)	Survival rate of zirconium abutments - 98.3% (single) - 99.2% (pontic without multi-units) - 96.1% (pontic with multi-units) Complication rate - 19.7% (single) - 3.9% (pontic without multi-units) - 3.8% (pontic with multi-units)	Zirconium abutments have an excellent long-term survival rate. For the restoration of a single posterior tooth, further studies are required.
Kim et al. [15]	Retrospective cohort	65/158/85	Anterior/premolar/molar	Single crown/splint crown/bridge	Alumina-toughened zirconium abutment (ZirAce)	78 months (60.9–117.5)	Zirconium fracture, screw fracture, and screw loosening were not observed for 5 years. Restoration success rate: 95.3%	Complications occurred after 5 years. The 5-year use is stable.
Bae et al. [16]	Prospective cohort	17/37	Maxilla mandible	Not mentioned	Zirconium/alumina composite abutment	12 months	No abutment Mean alveolar loss - Maxilla: 0.56 ± 0.26 - Mandible: 0.68 ± 0.30 Histological examination - Junctional epithelium Height: 2.09 mm Width: 0.51 mm	The zirconium alumina composite abutment is clinically stable.

#### Evidence level 4

A summary of six domestic kinds of literature with evidence level 4 is presented in Table 4. Evidence for the esthetic stability of zirconium abutments was shown in five kinds of literature out of six case reports with evidence level 4.

**Table 4:** Summary of domestic studies with evidence level 4

Author	Study type	Implant location	Abutment	F/U period	Outcome
Kim et al. [35]	Case report	#11	Customized zirconium myplant	Not mentioned	Interdental papillar 100% filled The shape and the color are harmonious. Functionally and esthetically satisfactory
		#21	Customized zirconium myplant	6 months	Harmonious with the adjacent teeth
		#11	Customized zirconium myplant	Not mentioned	Not mentioned
Byeon et al. [31]	Case report	#21	Not mentioned	2 months after installing the prosthesis	Esthetic, oral hygiene maintained
Lee et al. [32]	Case report	#23	Customized zirconium abutment	6 months after installing the prosthesis	Stable
Kim et al. [33]	Case report	#11 #21	Ready-made zirconium abutment (Osstem Korea)	10 months after treatment	Stable
Byeon et al. [36]	Case report	#11	Not mentioned	Not mentioned	Not mentioned
Yun et al. [37]	Case report	#21	Not mentioned	Not mentioned	Esthetic, oral hygiene maintained

### International Literature

#### Evidence level 2

A summary of the outcomes of five international literature with evidence level 2 is presented in Table 5. The stability evidence of zirconium abutments derived from the international literature with evidence level 2 can be roughly classified into two kinds. First, zirconium abutments are mechanically, biologically, and esthetically stable in general. In 16 literature evaluated by Medeiros et al. using the systematic literature review method [17], four literature evaluated by Guess et al. [18, 19], five literature evaluated by Gomes et al. [19], eight literature evaluated by Nakamura et al. [20], and one literature evaluated by Linkevicius et al. [21], the mechanical, biological, and esthetic stability of zirconium abutments was shown. The stability of zirconium abutments for long-term use, however, cannot be ensured. Medeiros et al. summarized 16 literature and revealed that the outcomes about the biological stability of zirconium abutments were varied [17]. Second, the stability of zirconium appeared to be positive within the short-term (4-year) clinical observation period. Four review literature, all reported that zirconium abutments are stable in the short term (within 5 years), but it is impossible to conclude the long-term stability of zirconium abutments due to the lack of long-term clinical studies.

**Table 5:** Summary of international studies with evidence level 2

Author	No. of studies (clinical studies)	Outcome	Conclusion
Medeiros et al. [17]	16	1. The zirconium abutments showed excellent soft-tissue reactions (3 studies). 2. The gingival recession increased in the zirconium abutments (1 study). 3. No biological difference was observed between the titanium and zirconium abutments (9 studies). 4. The zirconium abutments provided the gingival contour and anatomical shape of the natural teeth as well as have excellent esthetic features (3 studies).	1. Zirconium abutments are recommended for the anterior teeth. 2. Long-term studies are required to evaluate biological reactions.
Guess et al. [18]	18 (4)	The survival rate of the zirconium abutments was 100% (F/U period: 6 months-4 years).	As there are limited clinical data on zirconium abutments, their routine use in dental clinics is not recommended.
Gomes et al. [19]	20 (5)	1. The survival rate of the zirconium abutments was good. 2. The titanium abutments had more bone resorption than the zirconium abutments. 3. The zirconium abutments were esthetically and functionally stable. 4. No zirconium abutment fracture was observed for four years. 5. The zirconium abutments had less bacteria accumulation than the titanium abutments.	More studies on the long-term clinical success of zirconium abutments are required.
Nakamura et al. [20]	25 (8)	1. The zirconium abutments were acceptable for anterior teeth in the biological and mechanical aspects. 2. Zirconium had less early plaque accumulation than titanium.	The zirconium abutment has the potential to be used as a dental implant abutment material.
Linkevicius et al. [21]	9 (1)	Titanium abutments do not maintain a higher bone level than gold, aluminum oxide, and zirconium abutments.	Due to the lack of clinical studies, the stability of zirconium abutments cannot be determined.

#### Evidence level 3

In Table 6, the outcomes of seven international literature with evidence level 3 are summarized. The representative literature falling under evidence level 3 include cohort studies conducted by Zembic and Sailer [3, 6]. The studies conducted by Zembic and Sailer reported the survival rate of the single crown using 20 titanium abutments and 20 zirconium abutments in 40 patients [3, 6]. Additionally, the probing pocket depth, plaque control record, bleeding on probing, and bone level on radiography were compared with those of the opposite tooth. The stability evidence of zirconium abutments derived from international literature with evidence level 3 is roughly classified into three kinds.

**Table 6:** Summary of international studies with evidence level 3

Author	Study type	Patient/implant	Implant location	Prosthesis type	Abutment	F/U period	Outcome	Conclusion
Zembic et al. [3]	Prospective cohort	18/40	Canine, premolar, molar	Single crown	Zirconium, titanium	5.6 years (4.5–6.3)	1. Prosthesis survival rate: 100% 2. No significant difference in PD, PCR, BOP, and BL	No statistically or clinically significant difference in the 5-year survival rate
Van Brakel et al. [23]	Case-control study (histological examination)	22/44	Mandible (canine region)	No prosthesis	Zirconium, titanium	3 months	1. No significant difference in the vascular density of the adjacent tissues 2. No significant difference in the inflammation level	1. No difference in the soft-tissue health 2. No difference in the biological reactions
Bressan et al. [38]	Case-control study (The thickness and color of the soft tissue were measured.)	22/22	Maxilla anterior	Single crown	Gold, zirconium, titanium (all customized)	Not mentioned	1. The color of soft tissue around the implant is significantly different from that of the opposite tooth. 2. The titanium abutments showed a significantly greater color difference in soft tissue compared with gold or zirconium abutments. 3. The information on the difference in the soft-tissue thickness and the color difference cannot prove their correlations.	1. The color of the soft tissue around the implant is different from that of natural teeth regardless of the abutment materials. 2. The titanium abutments show a significantly greater color difference in the soft tissue compared with the gold or zirconium abutments. 3. The thickness of the soft tissue around the implant is not an important element in the effect of the abutment on the soft-tissue color.
Van Brakel et al. [22]	Case-control study	22/44	Mandible (canine region)	No prosthesis	Zirconium, titanium	3 months	1. In the two abutments, similar levels of bacteria were detected. 2. No significant difference in the soft-tissue condition around the implant	No difference in the soft-tissue health
Zembic et al. [7]	Prospective	18/40	Canine, premolar, molar	Single crown	Zirconium, titanium	36 months (31.5–53.3)	1. No significant difference in PD, PCR, BOP, and BL 2. The two abutments showed biological stability. 3. The degree of gingival discoloration in the two abutments was similar to that of the gingiva of natural teeth.	The zirconium and titanium abutments had the same survival rate and mechanical, biological, and esthetic outcomes during the 3-year follow-up period.
Sailer et al. [6]	Prospective cohort	22/40	Canine, premolar, molar	Single crown	Zirconium, titanium	12.6 months (± 2.7)	1. Prosthesis survival rate: 100% 2. No difference in PD, PL, and BOP 3. The colors of the soft tissue around the zirconium and titanium abutments are similar to the gingival color of natural teeth.	During the 1-year follow-up, the survival rates of the zirconium and titanium abutments are the same, and similar esthetic outcomes are shown.
Glausser et al. [2]	Prospective cohort	27/54	Incisor, canine, premolar	Single crown	Zirconium, titanium	48 months	The survival rate of the abutments: 100%	Zirconium abutments are very stable in supporting the single-tooth implant restoration at the anterior and premolar regions.

*PCR* plaque control record, *BOP* bleeding on probing, *PD* probing pocket depth, and *BL* bone level

First, zirconium abutments are mechanically stable within the 3- and 5-year follow-up periods. This study emphasized that the average 5-year follow-up period is longer than the follow-up periods of other studies. Second, zirconium abutments are stable for the restoration of a single tooth in the anterior and premolar regions. In the study of Glauser et al., single crowns using 54 zirconium abutments were observed for 4 years, and mechanical problems such as abutment fracture and abutment screw fracture, and marginal bone loss, were examined. During the average 49.2-month follow-up period, abutment fracture did not occur, and the mean marginal bone loss was reported to be 1.2 mm. Third, the esthetic stability of zirconium abutments cannot be determined [2]. Sailer et al. reported that there was no significant difference between zirconium and titanium abutments regarding their esthetic features. Fourth, zirconium abutments are biologically stable, but there is no significant difference between zirconium and titanium abutments regarding biological stability [6]. In the study of Van Brakel et al., in both zirconium and titanium, similar amounts of bacteria were detected, and the gingival health conditions were reported to be similar [22, 23]. After designing an experiment as described above, Van Brakel performed the histological examination. In the 3-month histological examination, no statistically significant differences in the vascular density and the inflammation level were found [23].

#### Evidence level 4

In Table 7, the outcomes of seven international literature with evidence level 4 are summarized. The study object of all the literature with evidence level 4 is the maxillary anterior region.

**Table 7:** Summary of international studies with evidence level 4

Author	Study type (no. of samples)	Implant location	Abutment	F/U period	Outcome
Lee et al. [24]	Case series (9)	Maxilla anterior	Zirconium abutment	52 weeks after installing the prosthesis	Esthetic, no abutment fracture or screw loosening
Aydin et al. [25]	Case report (1)	#22	Zirconium abutment	Six months after installing the prosthesis	No difference in PCR, BOP, PD, and BL
Kalman et al. [26]	Case report (1)	#22	Customized zirconium abutment (Nobel Procera)	Not mentioned	Esthetic
Wadhwani et al. [27]	Case report (1)	#12	Customized abutment (Straumann)	Not mentioned	Esthetic
Mahn et al. [28]	Case report (1)	#11	Customized abutment	Not mentioned	Esthetic
Schneider et al. [29]	Case report (1)	#21	Zirconium ART EASY abutment (Thommen Medical)	Not mentioned	Esthetic
Tan et al. [30]	Case report (1)	#21	Zirconium abutment (Astra Tech Inc., USA)	Not mentioned	Customized titanium abutment: excellent gingival contour, gray shade in the gingival area; zirconium abutment: no gray shade in the gingival area

*PCR* plaque control record, *BOP* bleeding on probing, *PD* probing pocket depth, and *BL* bone level

The following three results were derived from international literature with evidence level 4. First, zirconium abutments are mechanically stable. In the study conducted by Lee et al., when nine patients were observed for 52 weeks after early loading, no abutment fracture or abutment screw loosening occurred [24]. Second, zirconium abutments are biologically stable. Aydin et al. reported that there was no difference in the probing pocket depth, plaque control record, bleeding on probing, and bone level on radiography at the soft tissue around the zirconium implant 6 months after the prosthesis installation [25]. Third, zirconium abutments are generally esthetically stable. Kalman et al., Wadhwani et al., Mahn et al., and Schneider et al. reported the esthetic features in the maxillary anterior region [26–29]. However, Tan et al. reported that cast metal abutments have an excellent gingival contour and that zirconium abutment has an excellent gingival color when cast metal abutments are compared with ready-made zirconium abutments, indicating that zirconium abutments are not always superior to titanium abutments in terms of both the gingival contour and the color [30].

In this study, the domestic and international literature on the stability of zirconium abutments were analyzed, with a focus on evidence-based dentistry. As a result, the following shortcomings were shown.

First, the studies on the stability of zirconium abutments conducted for longer than 5 years are not sufficient. Considering that complications of zirconium prosthesis occur more than 5 years after, long-term studies lasting for more than 5 years are required. The analysis results of the domestic literature showed that zirconium abutments are mechanically, biologically, and esthetically stable, but most of the said literature have evidence level 4, meaning that the level of scientific evidence presented by them is low. Additionally, the stability of zirconium abutments is limited to the 5-year follow-up period. The evidence levels of international literature range from level 2 to level 4, and the scientific-evidence levels are higher and more varied compared to those of domestic literature. Like domestic literature, however, international literature reports the mechanical stability of zirconium abutments to last for 4 years; thus, the scientific evidence for the long-term stability of zirconium abutments is not sufficient. Further studies on the long-term survival rate of zirconium abutments are also required in other countries, and biological measurement values and esthetic outcomes need to be suggested using objective figures in long-term clinical practice.

Second, the domestic and international literature supporting the esthetic stability of zirconium abutments are case reports with a low evidence level, and most of them report the analysis results in a subjective language and have a short follow-up period or do not report the follow-up period. Among the domestic literature, three literature (written by Byeon et al. [31], Lee et al. [32], and Kim et al. [33], respectively) reported the follow-up period, and among the international literature, only two literature (written by Lee et al. [24] and Aydin et al. [25], respectively) reported the follow-up period. The rest of the literature did not mention the follow-up period. For the literature that reported the follow-up period, the maximum follow-up period was 52 weeks, and the average follow-up period was 6 months; thus, it is difficult to ensure esthetic stability. The outcomes were expressed in subjective language, only as “It is esthetic” or “It is stable,” and no statistically significant objective data were provided. Therefore, the conclusion that zirconium abutments are preferred to titanium abutments for an esthetically superior outcome does not have sufficient scientific evidence. To provide a scientific basis for the esthetic stability of zirconium abutments, the gingival color change, etc. need to be expressed objectively through the standardization of figures, using a tool such as a spectrophotometer. The esthetic stability must be evaluated after a more than 5-year-long observation after prosthesis installation.

## Conclusions

Zirconium abutments have a high survival rate regardless of implant location and prosthesis type within the 5-year follow-up period. Zirconium abutments are biologically stable. The evidence regarding the esthetic stability of zirconium abutments is not sufficient. Zirconium abutments are mechanically stable within the 5-year follow-up period. The long-term stability of zirconium abutments after 5 years cannot be determined. Therefore, clinical studies that investigate the long-term stability of zirconium abutments are required.

## Abbreviations

BL: Bone level
BOP: Bleeding on probing
MFDS: Ministry of Food and Drug Safety, Republic of Korea
PCR: Plaque control record
PD: Probing pocket depth
RCTs: Randomized controlled trials
